# NaV1.1 and NaV1.6 selective compounds reduce the behavior phenotype and epileptiform activity in a novel zebrafish model for Dravet Syndrome

**DOI:** 10.1371/journal.pone.0219106

**Published:** 2020-03-05

**Authors:** Wout J. Weuring, Sakshi Singh, Linda Volkers, Martin B. Rook, Ruben H. van ‘t Slot, Marjolein Bosma, Marco Inserra, Irina Vetter, Nanda M. Verhoeven-Duif, Kees P. J. Braun, Mirko Rivara, Bobby P. C. Koeleman

**Affiliations:** 1 Department of Genetics, Center for Molecular Medicine, UMC Utrecht Brain Center, University Medical Center Utrecht, Utrecht University, Utrecht, The Netherlands; 2 Department of Cardiology, Laboratory of Experimental Cardiology, University Medical Centre Leiden, Leiden, the Netherlands; 3 Department of Medical Physiology, University Medical Centre Utrecht, Utrecht, the Netherlands; 4 Centre for Pain Research & School of Pharmacy, University of Queensland, Brisbane, Australia; 5 Department of Neurology, University Medical Centre Utrecht, Utrecht, the Netherlands; 6 Food and Drug Department, University of Parma, Parma, Italy; Georgia State University, UNITED STATES

## Abstract

Dravet syndrome is caused by dominant loss-of-function mutations in SCN1A which cause reduced activity of Nav1.1 leading to lack of neuronal inhibition. On the other hand, gain-of-function mutations in SCN8A can lead to a severe epileptic encephalopathy subtype by over activating Na_V_1.6 channels. These observations suggest that Nav1.1 and Nav1.6 represent two opposing sides of the neuronal balance between inhibition and activation. Here, we hypothesize that Dravet syndrome may be treated by either enhancing Nav1.1 or reducing Nav1.6 activity. To test this hypothesis we generated and characterized a novel DS zebrafish model and tested new compounds that selectively activate or inhibit the human Na_V_1.1 or Na_V_1.6 channel respectively. We used CRISPR/Cas9 to generate two separate *Scn1Lab* knockout lines as an alternative to previous zebrafish models generated by random mutagenesis or morpholino oligomers. Using an optimized locomotor assay, spontaneous burst movements were detected that were unique to *Scn1Lab* knockouts and disappear when introducing human SCN1A mRNA. Besides the behavioral phenotype, *Scn1Lab* knockouts show sudden, electrical discharges in the brain that indicate epileptic seizures in zebrafish. *Scn1Lab* knockouts showed increased sensitivity to the GABA antagonist pentylenetetrazole and a reduction in whole organism GABA levels. Drug screenings further validated a Dravet syndrome phenotype. We tested the Na_V_1.1 activator AA43279 and two novel Na_V_1.6 inhibitors MV1369 and MV1312 in the *Scn1Lab* knockouts. Both type of compounds significantly reduced the number of spontaneous burst movements and seizure activity. Our results show that selective inhibition of Na_V_1.6 could be just as efficient as selective activation of Na_V_1.1 and these approaches could prove to be novel potential treatment strategies for Dravet syndrome and other (genetic) epilepsies. Compounds tested in zebrafish however, should always be further validated in other model systems for efficacy in mammals and to screen for potential side effects.

## Introduction

Dravet syndrome (DS), previously known as severe myoclonic epilepsy of infancy (SMEI), is a severe form of epilepsy for which current medication strategies remain largely inefficient. Promising new drugs that act on the serotonin pathway such as Fenfluramine (FA), show efficacy in reducing seizures in 50% to 90% of the patients [1]. However, drug side effects may still limit their use, underscoring the need for further drug discovery.

Of all DS patients, 95% carry a *de novo* heterozygous mutation in *SCN1A* [2], which encodes the pore forming α-subunit of neuronal voltage gated sodium channel (VGSC) type 1 (Na_V_1.1). Na_V_1.1 ion channels are the primary Na^+^ channels in GABAergic interneurons [3] [4] and induce the fast depolarization of neuronal membranes during action potential initiation. The majority of *SCN1A* mutations in DS are loss-of-function (LOF) mutations resulting in dysfunctional Na_V_1.1 channels, or reduced Na_V_1.1 expression [5, 6]. Consequently, excitability and action potential amplitude of interneurons are attenuated leading to reduced GABA release [7].

Another brain VGSC subtype, Na_V_1.6 is one of the two main sodium channels expressed in pyramidal neurons, which are responsible for excitatory signals via glutamate excretion [8]. *SCN8A*, which encodes the Na_V_1.6 α-subunit is also related to epilepsy and approximately 100 mutations have been reported in patients with severe Early Infantile Epileptic Encephalopathy subtype 13 (EIEE13). Unlike the clear LOF mutations in *SCN1A*, the majority of the functionally tested *SCN8A* mutations result in gain-of-function (GOF) of Na_V_1.6 [9]. GOF mutations in Na_V_1.6 cause channel hyperactivity due to augmented excitability and firing rates of pyramidal cells concurrent with an increase in glutamate release.

This disease mechanism is reflected by the therapeutic response of VGSC blockers. Various clinical reports have shown that *SCN8A*-related epilepsy patients benefit from VGSC blockers [10, 11], contrasting their inefficacy, or even detrimental effects in DS [12]. These observations indicate that Na_V_1.1 and Na_V_1.6 represent two opposing sides of the neuronal balance between inhibition and activation. We hypothesize that LOF mutations in *SCN1A* have a major negative effect on neuronal inhibition via hypo- activity of inhibitory interneurons, shifting the balance to neuronal hyperactivity. In contrast, GOF *SCN8A* mutations cause increased activity of excitatory pyramidal neurons, also shifting the VGSC-related balance towards neuronal hyperactivity. This model suggests that either selective activation of Na_V_1.1 or selective inhibition of Na_V_1.6 could be a therapeutic approach in the treatment of both DS and *SCN8A*-related epilepsy.

The therapeutic effect of Na_V_1.1 activation was previously shown in DS mice using spider venom peptide Hm1a that led to a reduction of seizures and mortality [13]. In another study using a different mouse strain, Hm1a was found to be lethal at picomolar doses within two hours [14], indicating that safety and administration needs to be further studied. Another Na_V_1.1 activator is the chemically synthesized small molecule AA43279, which showed anti-convulsant properties *in-vivo* but has not been tested in an animal model for DS. In comparison with Hm1a, AA43279 is less selective for Na_V_1.1, indicating it could activate other Na_V_ subtypes at lower concentrations. Nevertheless, AA43279 did not show lethality or sedative and ataxic side-effects at a concentration of 300mg/kg in mice [15].

Inhibition of Na_V_1.6 in DS was previously mimicked by introducing an *SCN8A* missense mutation in DS mice, which reduced seizure susceptibility and increased their lifespan [16]. Compounds that block Na_V_1.6 selectively have not been published to date, with the only exception a TTX metabolite for which the sensitivity of Na_V_1.1 is unknown [17].

To test if both Na_V_1.1 agonists and Na_V_1.6 antagonists could be beneficial in the treatment of DS we generated a novel DS zebrafish model by knocking out the *Scn1Lab* gene using CRISPR/Cas9. In humans, most of the mutations that cause DS are severe truncating mutations, while mild missense mutations are observed in patients with a milder epileptic phenotype [18]. To mimic the genetic architecture of DS in human patients as best possible, the animal model should display a 50% haploinsufficiency of *SCN1A*. Zebrafish likely carry two orthologues for the human *SCN1A* gene; *Scn1Laa* and *Scn1Lab*. While the expression of these genes does not overlap at embryonic- but only at larval stages [19, 20], they have a shared functional role in epilepsy [21]. Since 2010, three other *Scn1Lab* zebrafish models have been introduced, generated by N-ethyl-N-nitrosourea (ENU) mutagenesis [19, 22] or morpholino antisense oligomers (MO) [23]. All three display an epileptic phenotype that includes hyperactivity and epileptiform activity recorded from the brain. Nevertheless, CRISPR/Cas9 is currently the most efficient technique to specifically disrupt the gene that is targeted, unlike ENU mutagenesis [24], and acts on the DNA rather than protein as with MO based approaches [25, 26] allowing the generation of animal models that better mimic the genetic architecture of disease. As an improvement to previous zebrafish DS models, we generated two separate zebrafish strains with unique truncating mutations in *Scn1Lab* using CRISPR/Cas9.

The epileptic phenotype and drug response in zebrafish larvae can be measured by quantifying high velocity burst movements which are indicative of epileptic seizures in fish. The effect of anti-epileptic drugs on this unique behavior phenotype was found to be well correlated with reducing the number of spikes recorded from the zebrafish brain using a local field potential (LFP) set-up [27]. LFP spike events, however should not be confused with spikes of single neurons, but instead indicate the synchronized activity of large numbers of cells [28]. We performed LFP recordings in combination with a behavior essay to establish the initial phenotype in *Scn1Lab* knockouts, but used the locomotor assay as a single read-out on previously tested drugs as a validation for our model. After molecular validation, we tested Na_V_1.1 agonist AA43279 and two novel Na_V_1.6 channel antagonists, MV1369 and MV1312. Our results show that selective targeting of Na_V_1.1 or Na_V_1.6 ion channels reduced both the burst movement phenotype and epileptiform events in *Scn1Lab* knockout zebrafish, indicating a restoration in neuronal signaling.

## Results

### Generation of *Scn1Lab* knockout zebrafish

**Fig 1:**
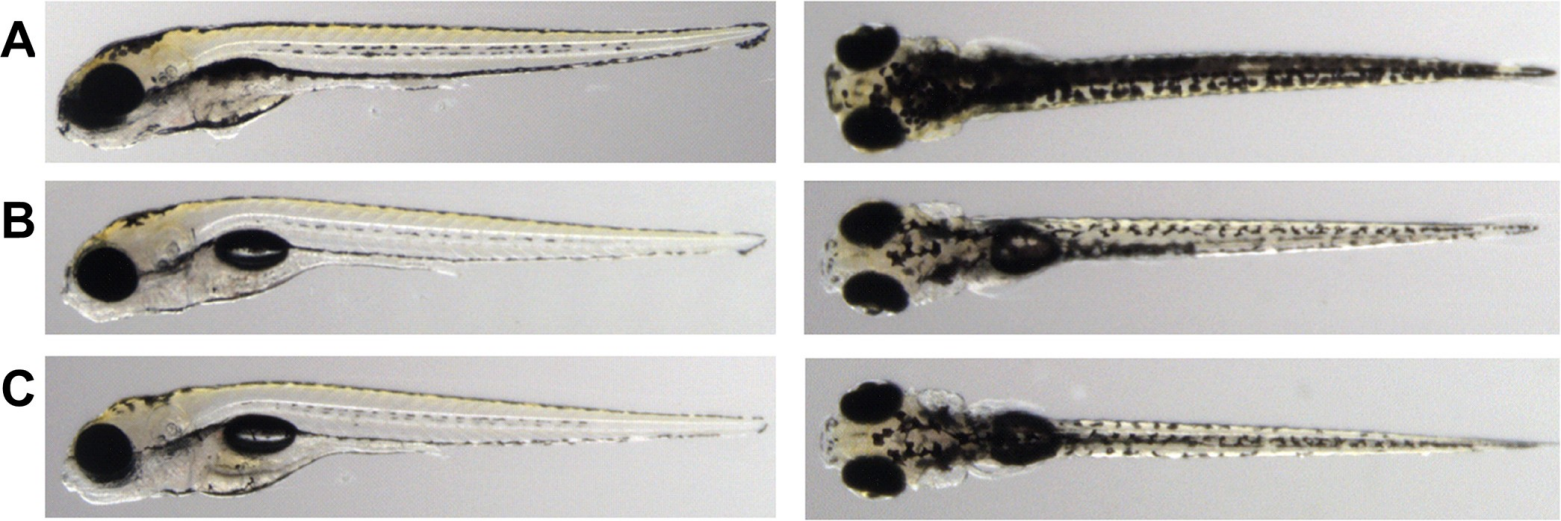
Morphology of the *Scn1Lab* knockout. A) 5 dpf *Scn1Lab* homozygous knockout larvae show hyperpigmentation and the absence of an inflated swim-bladder. These morphological defects are absent in heterozygous (B) or wildtype (C) zebrafish larvae.

Heterozygous and homozygous *Scn1Lab* knockout zebrafish were generated using CRISPR/Cas9. A 13 bp deletion was created in *Scn1Lab* exon 10, generating a STOP codon on position 474, truncating the *Scn1Lab* protein (S1A–S1C Fig). From here on, *Scn1Lab* knockout indicates 5 days post fertilization (dpf) larvae carrying the homozygous 13 bp deletion in *Scn1Lab*. A second allele, carrying a 5 bp deletion in exon 10, leading to a STOP codon on position 487 was generated in parallel to confirm the knockout phenotype (S1B Fig). To validate Cas9’ specificity in DNA editing, potential off-target regions were sequenced. No off-target editing of Cas9 was observed (S2 Fig). To verify the presence of the genomic deletion at transcription level, cDNA was sequenced, resulting in detection of the deletion (S3 Fig). *Scn1Lab* knockouts showed a similar morphological phenotype as observed in previous *Scn1Lab* knock-down models [23, 19, 21] including hyper-pigmentation and the absence of an inflated swim bladder (Fig 1A). Heterozygous knockout *Scn1Lab* larvae showed no apparent morphological difference compared to wildtype zebrafish (Fig 1B and 1C). As a control for the morphological differences of *ScnLab* knockouts, we generated *nisb-WT* control zebrafish [23] that lack an inflated swim bladder (S4 Fig).

### Epileptic phenotype of *Scn1Lab* knockouts

**Fig 2:**
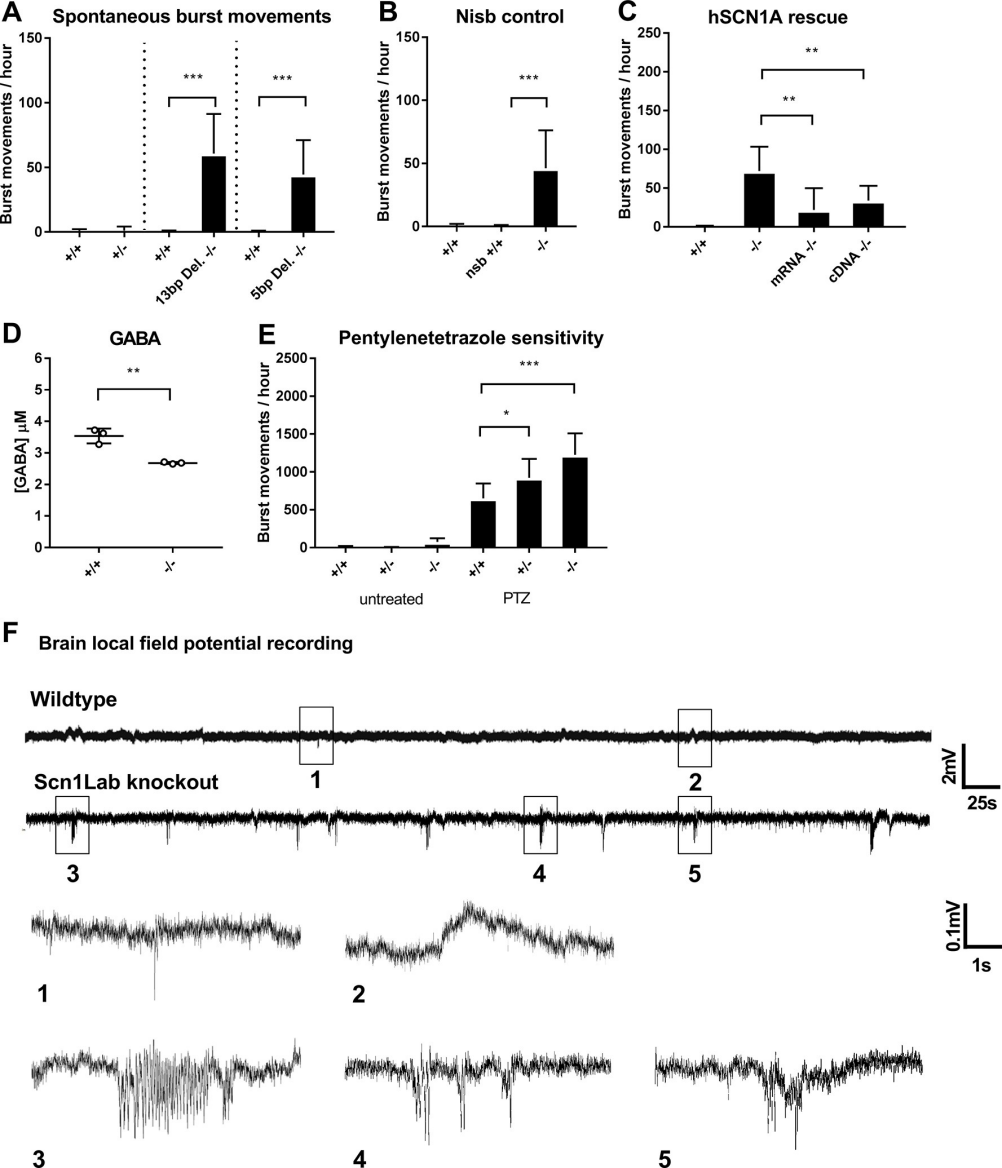
A) Spontaneous burst movements quantified in two knockout lines using the locomotor burst movement assay B) Burst movements are unique to *Scn1lab* knockouts, and not caused by the absence of an inflated swimming bladder C) The burst movement phenotype of *Scn1lab* knockouts is partially rescued when human SCN1A, either mRNA or cDNA is introduced D) *Scn1Lab* knockouts show a reduction in free GABA levels E) Both heterozygote and homozygous *Scn1lab* knockouts show sensitivity to exposure of 5mM pentylenetetrazole F) Representative non-invasive local field potential recordings from the brain of wildtype and *Scn1Lab* knockout zebrafish (n = 3). Only two types of signals can be detected in wildtype embryos: low amplitude waves and very occasional sharp single spikes from an otherwise straight and silent baseline (1 and 2). In *Scn1Lab* knockouts, at least three types of signal differ from wildtype recordings including trains of biphasic spikes lasting seconds (3) and several short spike events (4 and 5) which resembles epileptiform activity. *Error bars = S*.*D*. *(-/-) = Scn1Lab knockout*, locomotor assay n = 12 per group * <0.05 **<0.005 ***<0.*0005*.

*Scn1Lab* knockouts showed behavior comparable to previously described *Scn1Lab* knock-down models [23, 19, 21] including hyperactivity and high velocity burst movements (S1 Video and S5 Fig). Using optimized parameters, these specific, high velocity (>50mm/s) burst movements were separated from regular locomotor data to yield a burst movement assay. *Scn1Lab* knockouts showed a significantly higher number of spontaneous burst movements (Fig 2A). Using the *nisb-WT* control zebrafish, burst movements were found to be unique to *Scn1Lab* knockouts and not caused by the absence of an inflated swim bladder (Fig 2B). No spontaneous burst movements were observed in heterozygous knockout *Scn1Lab* larvae (Fig 2A). Using a LFP configuration for zebrafish larvae [29], abnormal brain activity was observed in *Scn1Lab* knockouts including multiple high frequency single-and poly spiking electrical discharges (Fig 2F). Epileptiform activity recorded from *Scn1Lab* knockouts resembles those observed in previous *Scn1Lab* knock-down zebrafish models [23, 19].

### Partial rescue of *Scn1Lab* knockout burst movements by human SCN1A

*Scn1Lab* is believed to be one of the two functional orthologues for human *SCN1A*, therefore we tested whether the spontaneous burst movement phenotype of *Scn1Lab* knockouts can be rescued by the introduction of human *SCN1A* in our model. Human *SCN1A* mRNA or cDNA was injected in one-cell stage *Scn1Lab* knockoutsleading to a partial rescue of burst movements. (Fig 2C).

### GABA reduction and sensitivity to pentylenetetrazole (PTZ) in *Scn1Lab* knockouts

To see if the GABAergic tone is disturbed in *Scn1Lab* knockouts, levels of free GABA were quantified in whole organism by Mass-spectrometry. *Scn1Lab* knockouts showed a statistically significant reduction of GABA, of approximately 25% (Fig 2D). Although the difference in GABA levels was low, the reduction indicates that *Scn1Lab* knockouts could be more susceptible to seizure inducing agents that act via the GABA pathway. A low dose of pentylenetetrazole (PTZ), a GABA antagonist frequently used as convulsant in animal studies was applied to *Scn1Lab* knockouts. Heterozygous and homozygous *Scn1Lab* knockouts showed a statistically significant increase in burst movements when exposed to 5mM PTZ, compared to wildtype zebrafish (Fig 2E).

### Pharmacological validation confirms a DS phenotype

**Fig 3:**
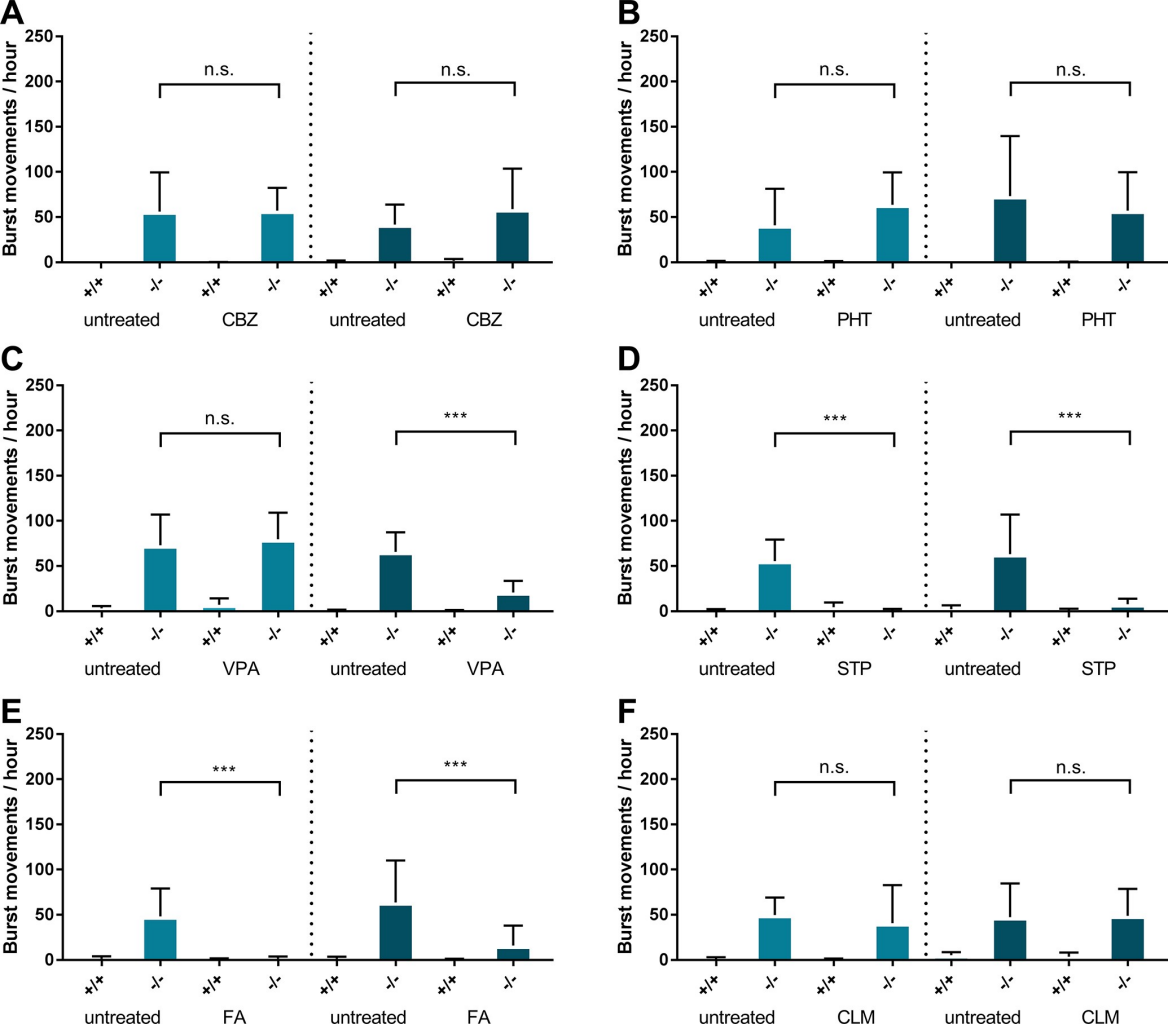
Pharmacologic validation of *Scn1Lab* knockouts by 60 minutes (light blue) or 18 hours (dark blue) exposure of anti-epileptic drugs A) 50μM Carbamazepine B) 100μM Phenytoin C) 100μM Sodium valproate D) 12.5μM Stiripentol E) 50μM Fenfluramine F) 50μM Clemizole. Dashed lines indicate a novel experimental plate with a seperate experimental group. *Error bars = S*.*D*., *(-/-) = Scn1Lab knockout*, n = 12 per group * <0.05 **<0.005 ***<0.*0005*.

Traditional VGSC blockers are known to be inefficient in *SCN1A* related epilepsies and can even sometimes aggravate seizures in Dravet syndrome, likely due to their limited Na_V_ subtype specificity. To test if VGSC blockers alter the burst movement phenotype in our *Scn1Lab* knockouts, the anti-epileptic drugs (AEDs) Phenytoin (PHT) and Carbamazepine (CBZ) were applied using a short or long drug exposure time. No reduction in burst movements was observed in the *Scn1Lab* knockouts (Fig 3A and 3B) when exposed to PHT or CBZ, confirming the inefficacy of VGSC blockers in DS. Next we tested the anti-seizure effects of GABA enhancing drugs Valproic acid (sodium valproate, VPA) and Stiripentol (STP), which are effective treatments for recurrent seizures in DS. Locomotor burst movements were significantly reduced when *Scn1Lab* knockouts were exposed to STP (Fig 3D). Interestingly, VPA showed an effect only after long exposure confirming previous reports, but short exposure did not result in burst movement reduction (Fig 3C).

### Partial rescue of burst movements by serotonin pathway modulators

Fenfluramine (FA) has previously been discovered using the *Scn1Lab* morpholino knock-down model to be effective in reducing DS seizures [23] and is now shown to be equally effective in our *Scn1Lab* knockout (Fig 3E). Clemizole (CLM), an antihistamine that can also bind to the serotonin receptor in zebrafish, was discovered using the ENU generated *Scn1Lab* knock-down model to be effective in reducing DS seizures [19]. When exposed for 24hours to equal concentrations of CLM, toxicity was observed including body malformations and death (S6 Fig). When exposed to 50% of this dose, toxicity was present in 20% of the larvae. When testing the larvae that appeared healthy for a burst movement phenotype reduction, we observed no effect (Fig 3F).

### Na_V_1.1 selective- but not general VGSC activators reduces burst movements

**Fig 4:**
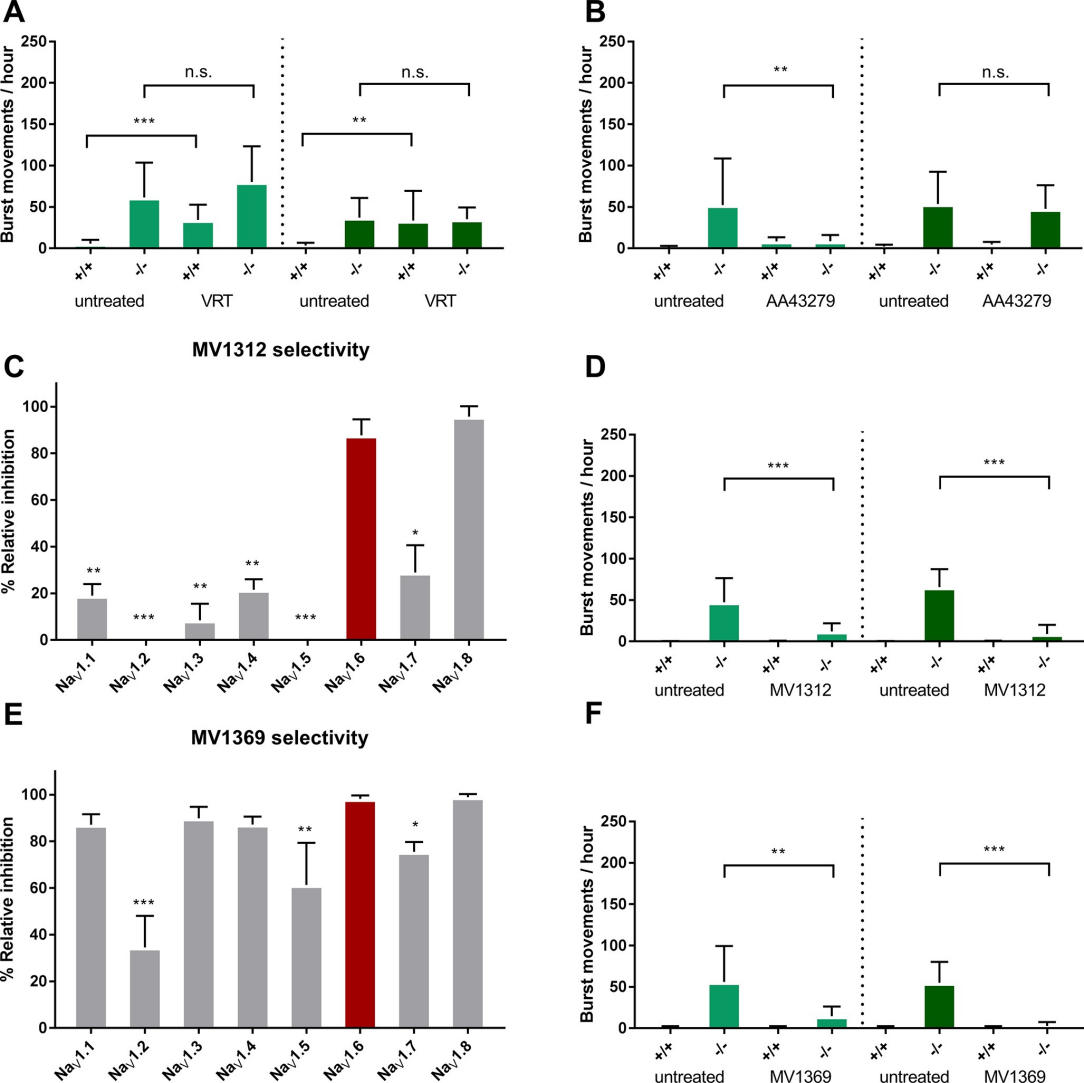
Characterization of novel compounds and their effect on *Scn1Lab* knockout burst movements by 60 minute (light green) or 18 hours (dark green) exposure A) 10μM Veratridine B) 5uM AA43279 C) MV1312 show blocking selectivity for Na_V_1.6 over the other human ion channel subtypes but not Na_V_1.8. D) 5μM MV1312 E) MV1369 shows blocking selectivity for Na_V_1.6 over Na_V_1.2, Na_V_1.5 and Na_V_1.7. F) 50μM MV1369. Dashed lines indicate a novel experimental plate with a seperate experimental group. *Error bars = S*.*D*., *(-/-) = Scn1Lab knockout*, *locomotor assays n = 12 per group*, *selectivity assays n = 3 per group*, * <0.05 **<0.005 ***<0.*0005*.

Wildtype zebrafish larvae exposed for a short or long incubation time to the general VGSC activator Veratridine (VRT) developed burst movements, confirming the convulsing effects of VRT in healthy control animals (Fig 4A). Interestingly, *Scn1Lab* knockouts revealed no additional increase, nor decrease of burst movements after being exposed to VRT (Fig 4A**)**. When exposed to Na_V_1.1 selective activator AA43279, the number of burst movements in *Scn1Lab* knockouts was significantly decreased (Fig 4B). This effect was only observed in the short, but not in the long exposure group. [15].

### Selectivity of MV1312 and MV1369 and efficacy in *Scn1Lab* knockouts

From a range of unpublished VGSC blocking compounds, several were tested for human Na_V_1.6 selectivity. MV1312 showed a 5–6 fold selectivity of Na_V_1.6 over Na_V_1.1–1.7, but comparable blocking affinity for Na_V_1.8 (Fig 4C). Na_V_1.8 is a peripheral nervous system ion channel involved in the sensation of pain, and blockage could lead to anti-nociception and pain treatment. As the selectivity over Na_V_1.1 is most important, we chose compound MV1312 to be a suitable candidate for further evaluation in our DS animal model. When exposed to 5μM MV1312, the number of burst movements was statistically significant reduced (Fig 4D), indicating a restoration of neuronal signaling in the epileptic brain. Another compound, named MV1369 showed less selectivity for sodium-channel subtype than MV1312. However, it did show higher selectivity for Na_V_1.6 as compared to Na_V_1.2 (Fig 4E). Interestingly, *Scn1Lab* larvae showed a reduction in burst movements when exposed to 50 μM MV1369, which is ten times higher than the effective concentration of MV1312 (Fig 4F). Despite the lower sodium-channel subtype selectivity, MV1369 reduces burst movements in the *Scn1Lab* knockout, which may suggest that the Scn1Laa gene can cover the function of more than one mammalian Na_V_-subtype.

### Reduced epileptiform activity in *Scn1Lab* knockouts exposed to MV1312 or AA43279

**Fig 5:**
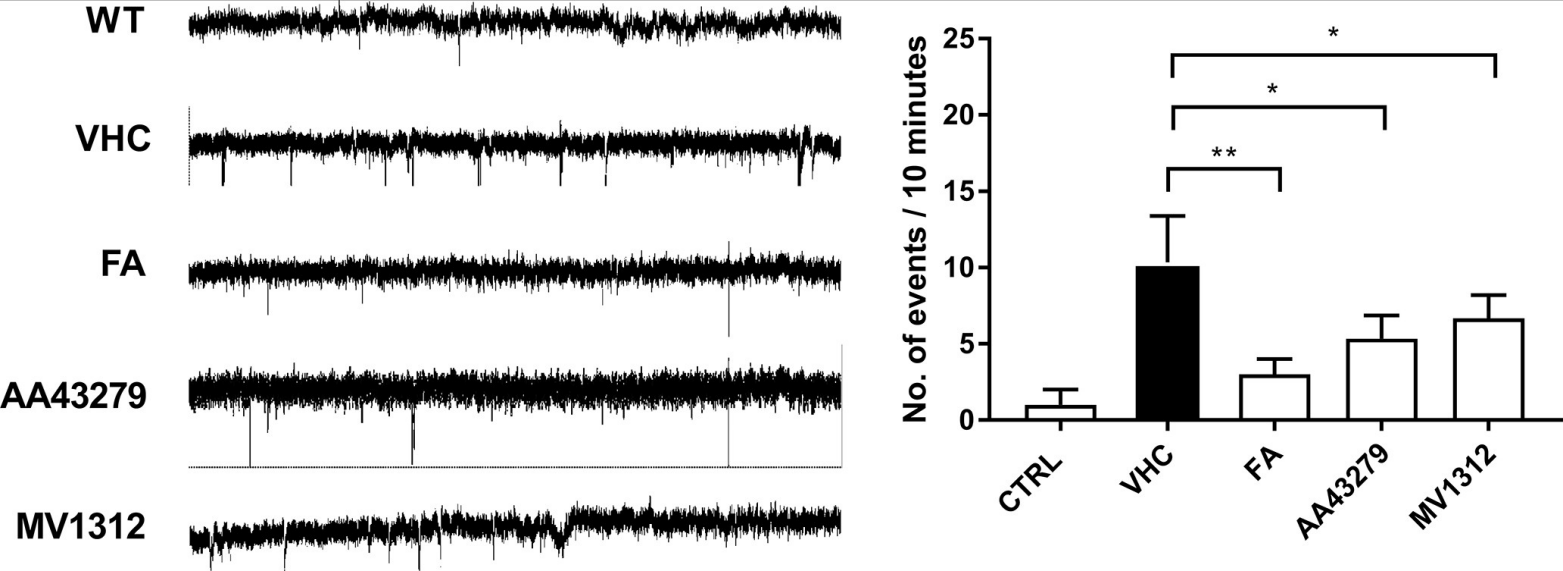
Epileptiform event scoring of wildtype or *Scn1Lab* knockout embryos exposed to FA, AA43279 or MV1312 for 60 minutes. Error bars = S.D n = 3 per group * <0.05 **<0.005.

To further validate the anti-seizure effects of VGSC selective compounds in our zebrafish model, we tested if the LFP recordings also showed a reduction of epileptiform events. To do so, we performed LFP recordings on wildtype or drug exposed *Scn1Lab* knockout larvae. In line with previous work, Fenfluramine effectively reduces the number of spontaneous electrical discharges of *Scn1Lab* knockouts (Fig 5). Also, both AA43279 and MV1312 reduce the number of epileptiform events (Fig 5), but the effect is less pronounced in comparison with FA. These observations are in line with the observed burst movement phenotype, which was reduced by FA to a baseline value whereas AA43279 and MV1312 showed a less strong effect. We did not correlate the reduction in burst movements to the reduction of LFP-recorded events, as they are counted differently. For example, a long trajectory of burst movements results in a higher score than short events, yet these could both be the correlated to a single epileptiform event.

## Discussion

Here, we present two CRISPR/Cas9 generated knockout zebrafish models for *SCN1A*-related epilepsies, including Dravet syndrome. The phenotype of *Scn1Lab* knockouts is characterized by spontaneous burst movements and sudden electrical discharges in the brain, a phenotype comparable to previous *Scn1Lab* knock-down and ENU-generated zebrafish models. Here, we used an optimized locomotor assay to extract burst movements from regular movement activity and found that the introduction of human *SCN1A* mRNA rescues this phenotype, indicating that *Scn1Lab* and SCN1A have a comparable function, at least during early development. *Scn1Lab* knockouts show a reduction in whole organism GABA levels, and are more sensitive to convulsions induced by GABA antagonist PTZ, compared to wildtype larvae. These results mimic haploinsufficiency of *SCN1A* in humans that likely affect local GABA levels, leading to the seizure susceptibility in DS patients. Using a local field potential setup for zebrafish larvae, we detect several signals from the brain that indicate seizure activity, including trains of polyspike discharges. By applying standard AEDs and DS specific drugs we observed a comparable pharmacological response as observed in the majority of DS patients, but also highlighted differences in drug response in comparison to previous *Scn1Lab* zebrafish models. Finally, we show that the Na_V_1.1 activator AA43279 and the Na_V_1.6 inhibiting compound MV1312 reduced the burst movement phenotype and the number of epileptiform events in *Scn1Lab* knockouts, indicating that selective ion channel subtype inhibition or activation could be beneficial in epilepsy.

In comparison to the other *Scn1Lab* zebrafish models, the *Scn1Lab* knockout also responds to VPA and STP but not to CBZ and PHT, mimicking the drug response in humans. FA was effective in the *Scn1Lab* morphant [23] and the *Scn1Lab* knockout, but not in the ENU generated *Scn1Lab* missense model (named didy^s552^ [22]. CLM did show effect in the ENU missense model [19, 21], but was shown to be ineffective in another study [22], which matches our result using the *Scn1Lab* knockout. There are several reasons for the differences in drug response. First and above all, different drug and solvent concentrations and drug incubation times have been used by different laboratories to characterize the drug response of *Scn1Lab* zebrafish models. The didy^s552^ has initially been characterized by exposing drugs in the millimolar range, using 7% DMSO for a 30, or 90 minute exposure time [19], while the MO model was exposed to micromolar concentrations, with 1% DMSO for 24 and 48 hour exposure time [23]. We were interested in concentrations of drugs and solvents [30] that do not cause toxicity regardless of the exposure time and therefore chose relatively low concentrations. Regarding drug exposure time, we propose that drug should be incubated at least 90 minutes to reach a peak metabolism as shown previously [31]. Interestingly, our results mimic the results of studies that use high drug and solvent concentrations, with the only exception being Clemizole. Second, all four *Scn1Lab* zebrafish models differ genetically and likely transcriptionally as they are generated by different techniques. ENU generated animal models likely carry more mutations on top of the one in the gene of interest, as ENU being a carcinogen, randomly mutates 1/100.000 basepairs approximately [24]. While the majority of these are intronic, it could be that they affect genes that regulate drug metabolism or perhaps expression of epilepsy-related genes. Third, the drug response could depend on Na_V_1.1b protein levels, which are not established in any of the *Scn1Lab* models due to the absence of suitable zebrafish antibodies. Future research using all four *Scn1Lab* zebrafish models, characterized in standardized experiments with safe solvent and drug concentrations could reveal why differences in drug response occur.

In our study, the non-selective VGSC activator Veratridine induced burst movements in wildtype zebrafish confirming its convulsing properties and suggesting that it likely has stronger effects on Nav1.6 inducing seizures, rather than increasing inhibition through Nav1.1. However, there was no burst movement increase detected in Veratridine exposed *Scn1Lab* knockouts, underlining that treatment of *SCN1A* haploinsufficiency could benefit from Na_V_ activators and the need for VGSC subtype selective compounds. AA43279 is a small molecule with reasonable selectivity for Na_V_1.1 over the other Na_V_ subtypes and was effective in our *Scn1Lab* knockouts. However, with lesser affinity AA43279 could also activate other Na_V_ subtypes, potentially leading to unwanted side effects. For example, off-target activation could lead to myotonia (Na_V_1.4), atrial fibrillation and cardiac arrest (Na_V_1.5), seizures (Na_V_1.2) or neuropathies (Na_V_1.7 and Na_V_1.8). For this reason, only compounds with high selectivity and efficacy at a very low dose are suitable candidates for translation to human patients. On the other hand, with future improved drug delivery systems such as viral particle-based or nanoparticles targeting the CNS specifically, off-target activation in the peripheral nervous system and other organs could be limited.

Inhibition, rather than activation of VGSC is a treatment method that might be preferential, especially when inhibition can be targeted to one or few channel subtypes only. Inhibitors of VGSC have been used for decades in epilepsy patients and although current VGSC blocking drugs such as carbamazepine and phenytoin are hardly selective, they have been proven safe in humans. Therefore, to assess whether inhibition of Nav1.6 can be a novel treatment strategy for DS, also applicable for epilepsy caused by SCN8A gain-of function mutations and perhaps also epilepsy in general, we tested two compounds that selectively inhibit Na_V_1.6 channels. MV1312 effectively rescued the burst movement phenotype and reduced epileptiform activity in our *Scn1Lab* knockouts comparable to AA43279 and Fenfluramine. For this reason, we believe that Na_V_1.6 selective inhibitors could be just as efficient as Na_V_1.1 selective activators and are potentially a safer choice in human patients. Another compound, MV1369 was found to be selective for Na_V_1.6 over Na_V_1.2, Na_V_1.5 and Na_V_1.7 and reduces the burst movement phenotype in *Scn1Lab* knockouts as well. There are several reasons why this type of compound is equally effective in the *Scn1Lab* knockout. First, compounds that are selective for human ion channel proteins, could have a different effect when applied to animal models, as the proteins are not identical to those in humans. Only minor differences on nucleotide level could allow, or limit proper binding of compounds in the protein binding site. Second, not all VGSC genes are evolutionary conserved in zebrafish, but only 6 other ion channel genes are known to exist beside *Scn1Laa* and *Scn1Lab*: *Scn4aa/ab*, *Scn5Laa/ab* and *Scn8aa/ab* [20]. While the function of these genes is not yet fully understood, it is clear that the absence of the full spectrum of Na_V_ subtypes in zebrafish limits measurable side-effects. As *Scn1Lab* is knocked out in our model, the supposed remaining inhibitory neurotransmission is regulated by *Scn1Laa*, highlighted by the efficacy of the Na_V_1.1 activator AA43279. However, it could very well be that *Scn1Laa* is only a partial functional duplicant of *Scn1Lab*, and carries a shared function and protein structure of *SCN1A*, *SCN2A*, *SCN3A* and even *SCN9A* as proposed earlier [20]. Nevertheless, zebrafish orthologs for SCN8A are well conserved, reaching >85% DNA and protein identity, highlighting that Na_V_1.6 inhibitors could be properly tested, but results should be interpreted with caution. Therefore, novel compounds tested in zebrafish, should be always be further studied in other model systems, preferably human-derived. To further improve treatment of genetic epilepsy, and reach selective activation or inhibition with more potency and selectivity, drugs should be designed that act on the nucleotide level. As disorders such as DS have a genetic cause, their treatment should also act at the genome-or transcriptome level. For this reason we are looking forward to clinically relevant treatments based on small activating RNAs, CRISPR/a/i and others that act on the human *SCN1A* or *SCN8A* gene.

## Materials & methods

### Zebrafish maintenance & ethics statement

All animal experiments were conducted under the guidelines of the animal welfare committee of the Royal Netherlands Academy of Arts and Sciences (KNAW). Adult zebrafish (*Danio rerio*) were maintained and embryos raised and staged as previously described [32]. Adult zebrafish were maintained in 4.5-liter polyethylene tanks (Tecniplast) in an Aqua Schwarz holding system (Göttingen) supplied continuously with circulating UV treated filtered tap water, which was exchanged for 10–30% daily. Average water properties were: Nitrit 0,095mg/L, Nitrat 16,7mg/L, Chloride and Ammonium 0mg/L, Hardness 9,8dH, pH 8,2, conductivity 460mS, Oxygen 6,85ppm and temperature 28,5C under cycles providing 14 hrs of light and 10 hrs of dark (14:10 LD; lights on 9 AM; lights off 11 PM). For all experiments described, larvae of 5 dpf were used. For imaging, larvae were embedded in 2% low-melting point agarose prepared with E3 medium.

### sgRNA design and Cas9 preparation

Gene specific guide RNAs (sgRNAs) were designed targeting *Scn1Lab* exon 10, using CHOPCHOP [33] with an off-target binding cut-off of 4 or more base pair mismatches. sgRNA oligonucleotides were synthesized according to previously described methods [34]. Oligos are listed in S1 Table. Capped Cas9 mRNA was created by *in vitro* transcription using Thermo Fisher mMESSAGE mMACHINE^™^ SP6 Transcription Kit from pCS2-nls-zCas9-nls (Addgene#47929).

### CRISPR/Cas9-sgRNA injections and genotyping

Fertilized eggs were injected with 2 nl of a solution containing 500ng *Cas9* mRNA, 150ng sgRNA and 0.2uL Phenol Red. sgRNAs targeting efficiency was tested by PCR amplifying the target region of 8 injected eggs at 2dpf. Primer sequences used for genotyping are listed in S1 Table. Injected embryos were raised to adult mosaic fishes. F0 founders were identified from week 10 by genotyping. F0 founders were outcrossed with wildtype fish to generate F1 embryos. F1 embryos were sampled for genotyping to confirm germline transmission of the mutation. The remaining F1 embryos were raised to adulthood and genotyped at week 10 by fin-clipping. Heterozygous knockouts carrying the same mutations were selected and crossed to raise the homozygous knockout F2 generation.

### CRISPR off-target assay

The gene-specific region including the protospacer adjacent motif (PAM) of the sgRNA was submitted to CCtop [35] to detect potential off-target sites. Five potential off-target sites with a maximum of four mismatches were selected for Sanger sequencing. Target sites, locations and the primers used for sequencing are included in S1 Table.

### GABA measurements

*Scn1Lab* knockout or wildtype larvae were pooled (n = 20) in eppendorf tubes in triplicates. Samples were centrifuged at 3500 rpm for 12 minutes at 4C° after which they were lysed in 500μL pre-chilled methanol using 0,5 mm zirconium oxide beads in a bullet blender. Samples were diluted 10 times and frozen at -80C° until day of analysis. A detailed Mass-spectrometry procedure be found in the Supplementary data.

### Locomotor assay

Locomotor experiments were performed under dark conditions at 28°C using 5dpf larvae placed in a flat bottom 48-well cell culture dish filled with 1mL E3/drug solution. Larvae were placed in single wells at 4 dpf to prevent stress from pipetting on the day of measurement. Movements were tracked in an automated tracking device (ZebraBox^™^; Viewpoint, Lyon, France) for 90 minutes, stacked in 10 minute bins, of which the first 30 minutes were removed as habituation time for the locomotor chamber. The final recording time for all locomotor experiments yielded one hour total. Threshold parameters of viewpoint software were freezing 1, sensitivity 8 and burst 50 resulting in a burst movement cut-off value of >50mm/s. Locomotor activity was quantified and analyzed by ZebraLab^™^ software by Viewpoint.

### hSCN1A Rescue

The SCN1A plasmid, which encodes the human neonatal Nav1.1 ion channel, was previously described [36, 37]. Capped hSCN1A mRNA was *in vitro* transcribed using Thermo Fisher mMESSAGE mMACHINE^™^ T3 Transcription Kit. For mRNA injections, 200ng/ul mRNA was injected in the yolk of one-cell stage zebrafish embryos.

### Local field potential recordings

LFP setup and concurrent recording settings were based on previous work [29]with slight modifications. In short, a silver wire carrying glass electrode connected to a high-impedance amplifier, was filled with 1mM NaCl. All larvae that were recorded were exposed to 20 μM D-Tubocurarine pentahydrate as a muscle relaxant for 10 minutes in order to reduce electro-mechanical artefacts caused by physical twitching. Next, a single larva was embedded in 1.5% low-melting-point agarose (Invitrogen) and the electrode was placed on top of the forebrain. Recordings were performed in current clamp mode using the DAGAN EX-1 amplifier, national instruments 6210 USB digitizer and WinEDR software. The following parameters were used: 3kHz low-pass filter, 0.3Hz high-pass filter, digital gain 10 and 10 μs sampling interval. Recordings were performed for ten minutes after a ten minute habituation time, and epileptiform events were scored manually.

### Synthesis of MV1312 (3) and MV1369 (6)

**Scheme 1:**
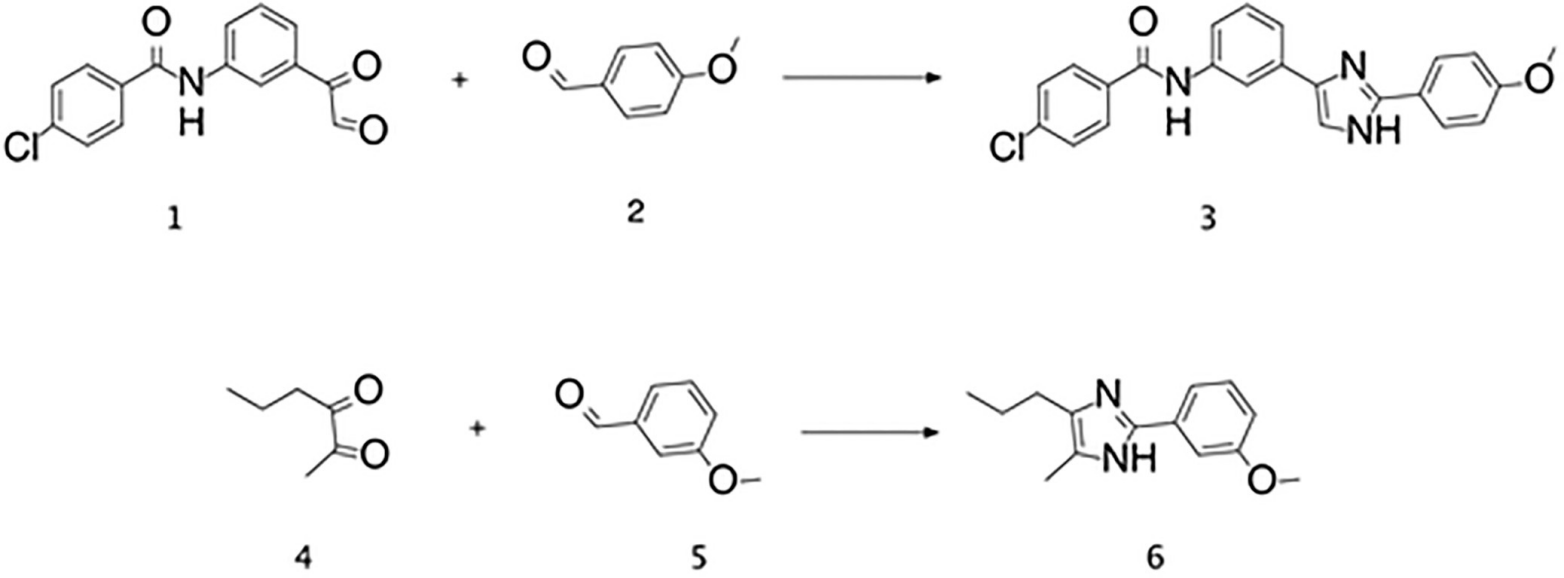
Reagents and conditions: 1 equiv of 2 and 5 with 4 equiv CH_3_COONH_4_ in 3.8 mL CH_3_OH; 1 equiv of 1 and 4 (respectively) in 3.5 mL CH_3_OH. Overnight rt.

Synthesis of MV1312 (4-Chloro-n-{3-[2-(4-methoxy-phenyl)-1h-imidazol-4-YL]-phenyl}-benzamide) and MV1369 (2-(3-methoxy-phenyl)-5-methyl-4-propyl-1h-imidazole) is depicted in Scheme 1. Details on the synthesis procedure can be found in the Supplementary data.

### Determination of compound selectivity

Activity of MV1312 and MV1369 at hNav1.1–1.8 was assessed using a fluorescent imaging plate reader (FLIPRTetra, molecular devices) membrane potential assay as previously described^55^. In brief, cell lines (HEK293 Nav1.1–1.8) were plated on 384-well black-walled imaging plates (Corning) at a density of 10 000–15 0000 cells per well 48 hours before loading with 20 μ L of red membrane potential dye (proprietary formulation) (Molecular Devices, Sunnyvale, CA). Cells were incubated with the membrane potential dye for 30 min at 37 °C before the addition of compounds by the FLIPR^Tetra^ system. After the addition of 100μM MV1312, fluorescence was measured (excitation 515–545 nm; emission: 565–625 nm) for a period of 5 min to determine the effects of the compounds alone. Following this 5 min exposure, 5–20 μM VRT was added and fluorescence was measured for a further 5 min. Data was recorded and converted to response over baseline using Screenworks 3.2.0.14.

### Drug treatment

E3/drug solutions (2X) were prepared in 0.8% DMSO at their maximum tolerated concentration (MTC) as calculated elsewhere [23, 19, 27]. In summary, the following MTCs were used: Carbamazepine (CBZ) 50μM, Phenytoin (PHT) 100μM, Valproic acid (VPA) 100μM, Stiripentol (STP) 12,5μM, Fenfluramine (FA) 50μM, Clemizole (CLM) 100μM, 200μM (both toxic) and 50μM. 500μL of AED solution was pipetted in each single well containing 500μL E3 generating a final DMSO concentration of 0.4%. VHC = 0.4% DMSO. For short incubation experiments larvae were incubated for 1 hour, for long incubation experiments, the incubation time was 18 hours. Compounds were ordered at Sigma-Aldrich.

### Determination of maximum tolerated concentration (MTC)

Compounds AA43279, MV1312 and MV1369 were tested for toxicity based on previously established methods [23] with slight modifications. In short; compounds were incubated in the bathing medium of 4 dpf larvae. After 24 hours, the following toxicity parameters were checked: touch response, loss of posture, body deformation and death. When none of these parameters were observed in any of the larvae tested, the concentration was regarded safe.

### Statistical analysis

Data was analyzed and plotted using Graphpad Prism 7.04. Locomotor data did not pass the D’agostino & Pearson normality test, therefore the non-parametric Mann-Whitney U-test was used for data analysis. Mass-spectrometry was normally distributed and was further analyzed using the student t-test. Compound selectivity and seizure event scoring data was normally distributed and therefore analyzed with a multi-comparison ANOVA. A *P*-value of <0.05 was considered significant.

## Supporting information

S1 File Ultra-performance liquid chromatography coupled to tandem mass spectroscopy (UPLC-MS/MS) experiments. (DOCX)

S2 File Synthesis of MV1312 and MV1369. (DOCX)

S1 Fig gDNA sequencing *Scn1Lab* knockouts. A and B) Sanger traces from the deletion sites at exon 10, showing a wildtype, heterozygous and homozygous knockout trace. Black bar: deleted base pairs Grey arrow: sequence trace directly after the deletion site. C) Graphical overview of Sanger traces showing the deletion site and their effect on the reading frame. Red triangle; Cas9 cut site (3 bases upstream of PAM) PAM: protospacer adjacent motif required for Cas9 binding (NGG).
(DOCX)

S2 Fig Off target sequencing. Following CCtop prediction software, five potential off-target sites for Cas9 were Sanger sequenced. None of the off-target sites were found edited.
(DOCX)

S3 Fig cDNA sequencing of *Scn1Lab* knockout. The cDNA of heterozygous *Scn1Lab* knockouts was Sanger sequenced to yield the deletion at transcription level.
(DOCX)

S4 Fig Non-inflated swimbladder wildtype zebrafish morphology. A) wildtype zebrafish submerged underwater to prevent swimbladder inflation B) *Scn1Lab* knockout zebrafish, also without inflated swim-bladder as a comparison.
(DOCX)

S5 Fig Raw locomotor data Scn1Lab knockout and wildtype zebrafish larvae. In white wildtype burst movements and actinteg units (overall movement activity), in black the same parameters plotted from Scn1Lab knockouts. Raw data is annotated in the table on the right side, each cell indicates a single larva. Error bar = S.D. * = p<0.05 ** = p<0.0005.
(DOCX)

S6 Fig Clemizole toxicity after long-term exposure. *Scn1Lab* embryos exposed to 100μM or 200μM clemizole showed toxicity after 24h incubation including malformations and death. 50μM Clemizole was used instead of 100μM for AED exposure experiments.
(DOCX)

S1 Table Oligos. (DOCX)

S1 Video (MP4)
