# Supplementary material for: NaV1.1 and NaV1.6 selective compounds reduce the behavior phenotype and epileptiform activity in a novel zebrafish model for Dravet Syndrome
Source: PLoS One. 2020 Mar 5;15(3):e0219106. doi: 10.1371/journal.pone.0219106 (PMC7058281; doi:10.1371/journal.pone.0219106)
Supplement: S1 File — (DOCX) [file pone.0219106.s001.docx]

**Ultra-performance liquid chromatography coupled to tandem mass spectroscopy (UPLC-MS/MS) experiments**

*Reagents*

GABA, ^2^H_6_-GABA, acetylchloride and ammoniumformiate were purchased from Sigma-Aldrich (Zwijndrecht, the Netherlands). Formic acid was purchased from VWR (Amsterdam, the Netherlands), Acetonitrile (ACN) UPLC-grade was purchased from Biosolve (Valkenswaard, the Netherlands) and butanol was purchased from Merck (Amsterdam, the Netherlands).

*Sample preparation*

Stock solutions of GABA and 2H6-GABA were prepared in milliQ-water (5 mmol/L), and were diluted to obtain working solutions of 1 µmol/L GABA and 1 µmol/L 2H6-GABA, respectively. For quantification of GABA, calibration curves were prepared to obtain concentrations of 0, 100, 200, 300, 400 and 500 nmol/L.

Before analysis, samples were thawed and vortexed. 100 µl sample was mixed with 20 µL 1 µmol/L 2H6-GABA after which 500 µL ACN was added. The samples were vortexed, centrifuged at 13000 rpm for 5 min at room temperature and the supernatant was transferred to a 96 wells-plate (Waters, Etten-Leur). The samples were evaporated to dryness under a stream of nitrogen at 40 °C. After addition of 100 µL 3M butylation reagent (consisting of 4:1 butanol and acetylchloride), the 96 wells-plate was placed on a shaker (Scientific Industries, New York, USA) for 1 minute and subsequently placed for 15 minutes at 60 °C. Next, the samples were evaporated to dryness under a stream of nitrogen at 40 °C. Samples were dissolved in 100 µl ACN, after which the 96-wells plate was placed at the shaker again for 1 minute. After this step, the samples were ready for UPLC-MS/MS analysis.

*Quality Control (QC)*

Two calibrators (QC-high and QC-low) were prepared for quality control by spiking a plasma sample to obtain GABA concentrations in the range of approximately one-third and two-third of the calibration curve.

*Performance characteristics*

Performance characteristics that were tested include carry-over, detection limit (LOD), limit of quantification (LOQ), linearity, within-run variation, between run variation, stability, analytical sensitivity, interferences and uncertainty of measurement. The uncertainty of measurement was defined as twice the between run variation. LOD was calculated as three times signal to noise (S/N)-ratio, while LOQ was calculated as ten times signal to noise (S/N)-ratio.

*Instrument*

The chromatographic separation for plasma was carried out on an Acquity UPLC BEH Amide column (2.1 mm x 100 mm, 1.7 µm particle size) including a Van Guard^TM^ UPLC BEH Amide pre-column (2.1 x 5 mm, 1.7 µm particle size) (Waters, Milford, USA) (See figure S1). The column was maintained at a temperature of 40 °C and the sample volume injected was 5 µL. Optimal chromatographic separation was achieved at a flow-rate of 0.4 ml/min using a isocratic gradient with 5% v/v ammoniumformiate (50 mM pH 3 and 95 % ACN). Total run time was 5 minutes. The column was coupled to the mass spectrometer. A Xevo-TQ MS triple quadrupole mass spectrometer with an electrospray ionization (ESI) source and an Acquity UPLC-system (Waters, Manchester, United Kingdom) were used. Masslynx software (v4.1; Waters, Manchester, United Kingdom) was used for instruments’ control and data acquisition. The mass spectrometer operated in ESI-positive mode, capillary voltage 0.5 kV, desolvation temperature 600 °C, source temperature 150 °C, cone gas flow 0L/h, desolvation gas flow was 700 L/h. Collision energy and cone voltage were optimized for GABA. The dwell time was set automatically. Positively singly charged ions [M + H]^+^ of GABA (m/z 160.2) and ^2^H_6_-GABA (m/z 166.2) were selected as parent ions for Collision Induced Dissociation (CID). The daughter ion m/z 87.0 and 93.0 were the most abundant ions for GABA and ^2^H_6_-GABA, respectively. For multiple reaction monitoring (MRM), the transitions m/z 160.2 🡪 87.0 (GABA) and m/z 166.2 🡪 93.0 (^2^H_6_-GABA) were measured.
