# Supplementary material for: NaV1.1 and NaV1.6 selective compounds reduce the behavior phenotype and epileptiform activity in a novel zebrafish model for Dravet Syndrome
Source: PLoS One. 2020 Mar 5;15(3):e0219106. doi: 10.1371/journal.pone.0219106 (PMC7058281; doi:10.1371/journal.pone.0219106)
Supplement: S2 File — (DOCX) [file pone.0219106.s002.docx]

**Synthesis of MV1312 and MV1369**

For MV1312 (3), to a mixture of p-anisaldehyde (0.7 mmol) and ammonium acetate (3.41 mmol) in methanol (3.5 mL) was added a solution of 4‐chloro‐N‐[3‐(2‐oxoacetyl)phenyl]benzamide (0.7 mmol) in methanol (3.8 mL). The reaction mixture was stirred overnight at room temperature, then the solvent was evaporated and the residue was partitioned between saturated aqueous NaHCO_3_ solution (20 mL) and ethyl acetate (20 mL). The organic phase was dried over Na_2_SO_4_ and the solvent was removed in vacuo. The isolation of MV1312 from the crude reaction mixture was obtained using flash chromatography on a silica gel column. The hydrochloride salt was prepared by treating the free base with an ethanolic HCl 5% w/w solution. The product was then crystallized from absolute ethanol/dry diethyl ether. 
Yield= 80%, mp (hydrochloride) 290–292 °C. 1H NMR (300 MHz, DMSO-d6): 10.59 (s, 1H), 8.36 (br s, 1H), 8.21 (d, 2H), 8.14 (s, 1H), 8.06 (d, 2H), 7.72 (m, 2H), 7.65 (m, 2H), 7.54 (t, 1H), 7.24 (d, 2H), 3.89 (s, 3H). MS (EI) 404 [M+]. Anal. Calcd for C_23_H_18_ClN_3_O_2_*HCl: C, 62.74; H, 4.35; N, 9.54. Found: C, 62.86; H, 4.52; N, 9.78.
For MV1369 (6), To a mixture of m-anisaldehyde (0.7 mmol) and ammonium acetate (3.41 mmol) in methanol (3.5 mL) was added a solution of hexane‐2,3‐dione (0.7 mmol) in methanol (3.8 mL). The reaction mixture was stirred overnight at room temperature, then the solvent was evaporated and the residue was partitioned between saturated aqueous NaHCO_3_ solution (20 mL) and ethyl acetate (20 mL). The organic phase was dried over Na_2_SO_4_ and the solvent was removed in vacuo. The isolation of MV1369 from the crude reaction mixture was obtained using flash chromatography on a silica gel column. The oxalate salt was prepared by treating the free base with a saturated ethanolic solution of oxalic acid. The product was then crystallized from absolute ethanol/dry diethyl ether. 
Yield = 68%, mp (oxalate) 130-133 °C. 1H NMR (300 MHz, DMSO-d6): 7.53 (m, 2H), 7.42 (t, 1H), 7.02 (d, 1H), 3.82 (s, 3H), 2.56 (t, 2H), 2.22 (t, 3H), 1.61 (m, 2H), 0.90 (t, 3H). MS (EI) 231 [M+]. Anal. Calcd for C_14_H_18_N_2_O*(COOH)_2_: C, 59.99; H, 6.29; N, 8.74. Found: C, 59.86; H, 6.52; N, 8.88.
