# Supplementary material for: NaV1.1 and NaV1.6 selective compounds reduce the behavior phenotype and epileptiform activity in a novel zebrafish model for Dravet Syndrome
Source: PLoS One. 2020 Mar 5;15(3):e0219106. doi: 10.1371/journal.pone.0219106 (PMC7058281; doi:10.1371/journal.pone.0219106)
Supplement: S1 Fig — A and B) Sanger traces from the deletion sites at exon 10, showing a wildtype, heterozygous and homozygous knockout trace. Black bar: deleted base pairs Grey arrow: sequence trace directly after the deletion site. C) Graphical overview of Sanger traces showing the deletion site and their effect on the reading frame. Red triangle; Cas9 cut site (3 bases upstream of PAM) PAM: protospacer adjacent motif required for Cas9 binding (NGG). (DOCX) [file pone.0219106.s003.docx]

**


S1 gDNA sequencing *Scn1Lab* knockouts** A and B) Sanger traces from the deletion sites at exon 10, showing a wildtype, heterozygous and homozygous knockout trace. Black bar: deleted base pairs Grey arrow: sequence trace directly after the deletion site. C) Graphical overview of Sanger traces showing the deletion site and their effect on the reading frame. Red triangle; Cas9 cut site (3 bases upstream of PAM) PAM: protospacer adjacent motif required for Cas9 binding (NGG).
