# Supplementary material for: NaV1.1 and NaV1.6 selective compounds reduce the behavior phenotype and epileptiform activity in a novel zebrafish model for Dravet Syndrome
Source: PLoS One. 2020 Mar 5;15(3):e0219106. doi: 10.1371/journal.pone.0219106 (PMC7058281; doi:10.1371/journal.pone.0219106)
Supplement: S2 Fig — Following CCtop prediction software, five potential off-target sites for Cas9 were Sanger sequenced. None of the off-target sites were found edited. (DOCX) [file pone.0219106.s004.docx]

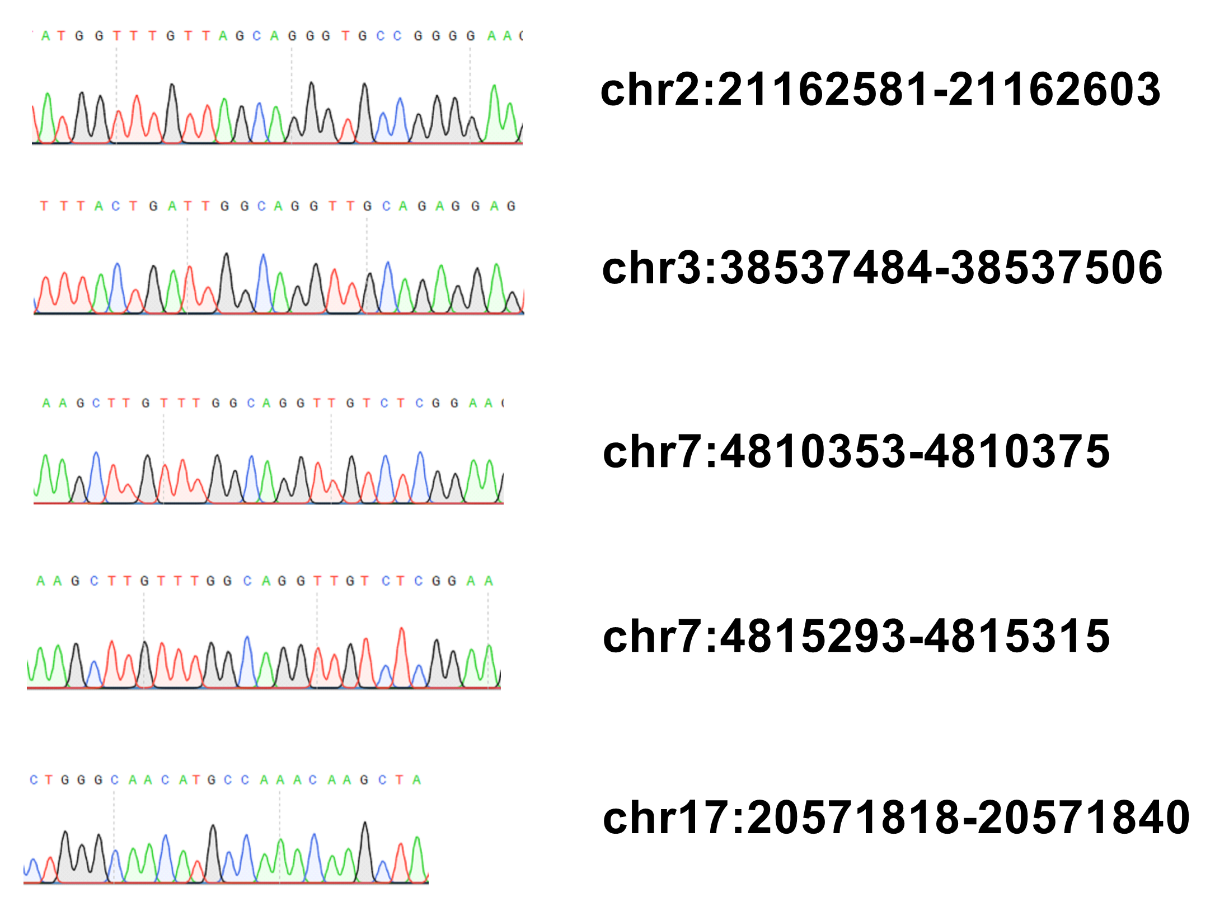


**S2 Off target sequencing** Following CCtop prediction software, five potential off-target sites for Cas9 were Sanger sequenced. None of the off-target sites were found edited.
