# Supplementary material for: NaV1.1 and NaV1.6 selective compounds reduce the behavior phenotype and epileptiform activity in a novel zebrafish model for Dravet Syndrome
Source: PLoS One. 2020 Mar 5;15(3):e0219106. doi: 10.1371/journal.pone.0219106 (PMC7058281; doi:10.1371/journal.pone.0219106)
Supplement: S3 Fig — The cDNA of heterozygous Scn1Lab knockouts was Sanger sequenced to yield the deletion at transcription level. (DOCX) [file pone.0219106.s005.docx]

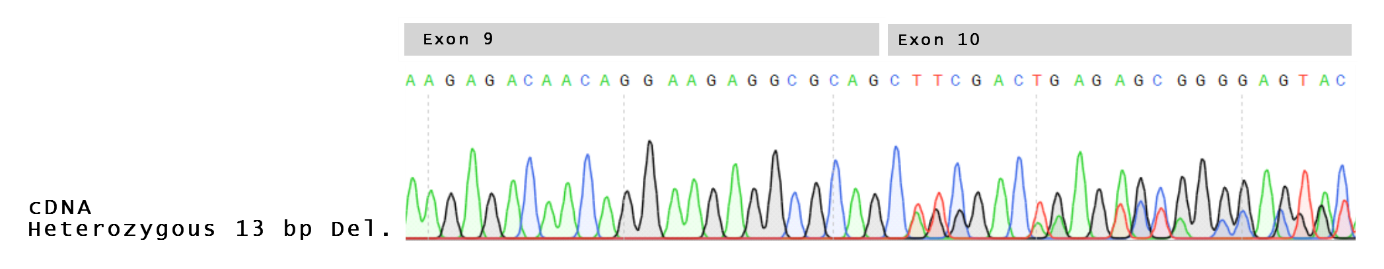


**S3 cDNA sequencing of *Scn1Lab* knockout** The cDNA of heterozygous *Scn1Lab* knockouts was Sanger sequenced to yield the deletion at transcription level.
