# Supplementary material for: NaV1.1 and NaV1.6 selective compounds reduce the behavior phenotype and epileptiform activity in a novel zebrafish model for Dravet Syndrome
Source: PLoS One. 2020 Mar 5;15(3):e0219106. doi: 10.1371/journal.pone.0219106 (PMC7058281; doi:10.1371/journal.pone.0219106)
Supplement: S4 Fig — A) wildtype zebrafish submerged underwater to prevent swimbladder inflation B) Scn1Lab knockout zebrafish, also without inflated swim-bladder as a comparison. (DOCX) [file pone.0219106.s006.docx]

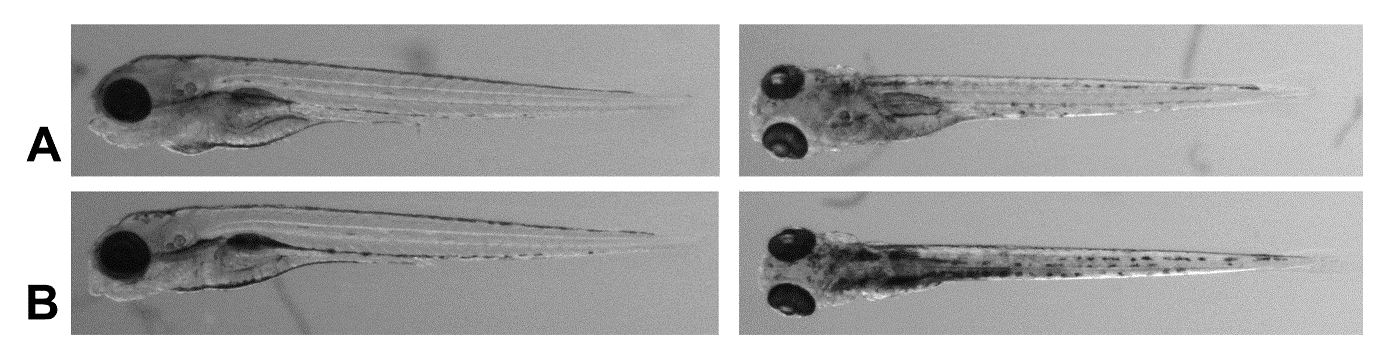


**S4 Non-inflated swimbladder wildtype zebrafish morphology** A) wildtype zebrafish submerged underwater to prevent swimbladder inflation B) *Scn1Lab* knockout zebrafish, also without inflated swim-bladder as a comparison.
