# Supplementary material for: NaV1.1 and NaV1.6 selective compounds reduce the behavior phenotype and epileptiform activity in a novel zebrafish model for Dravet Syndrome
Source: PLoS One. 2020 Mar 5;15(3):e0219106. doi: 10.1371/journal.pone.0219106 (PMC7058281; doi:10.1371/journal.pone.0219106)
Supplement: S5 Fig — In white wildtype burst movements and actinteg units (overall movement activity), in black the same parameters plotted from Scn1Lab knockouts. Raw data is annotated in the table on the right side, each cell indicates a single larva. Error bar = S.D. * = p<0.05 ** = p<0.0005. (DOCX) [file pone.0219106.s007.docx]

**S5 Raw locomotor data Scn1Lab knockout and wildtype zebrafish larvae** In white wildtype burst movements and actinteg units (overall movement activity), in black the same parameters plotted from Scn1Lab knockouts. Raw data is annotated in the table on the right side, each cell indicates a single larva. Error bar = S.D. *=p<0.05 **=p<0.0005
