# Supplementary material for: NaV1.1 and NaV1.6 selective compounds reduce the behavior phenotype and epileptiform activity in a novel zebrafish model for Dravet Syndrome
Source: PLoS One. 2020 Mar 5;15(3):e0219106. doi: 10.1371/journal.pone.0219106 (PMC7058281; doi:10.1371/journal.pone.0219106)
Supplement: S6 Fig — Scn1Lab embryos exposed to 100μM or 200μM clemizole showed toxicity after 24h incubation including malformations and death. 50μM Clemizole was used instead of 100μM for AED exposure experiments. (DOCX) [file pone.0219106.s008.docx]

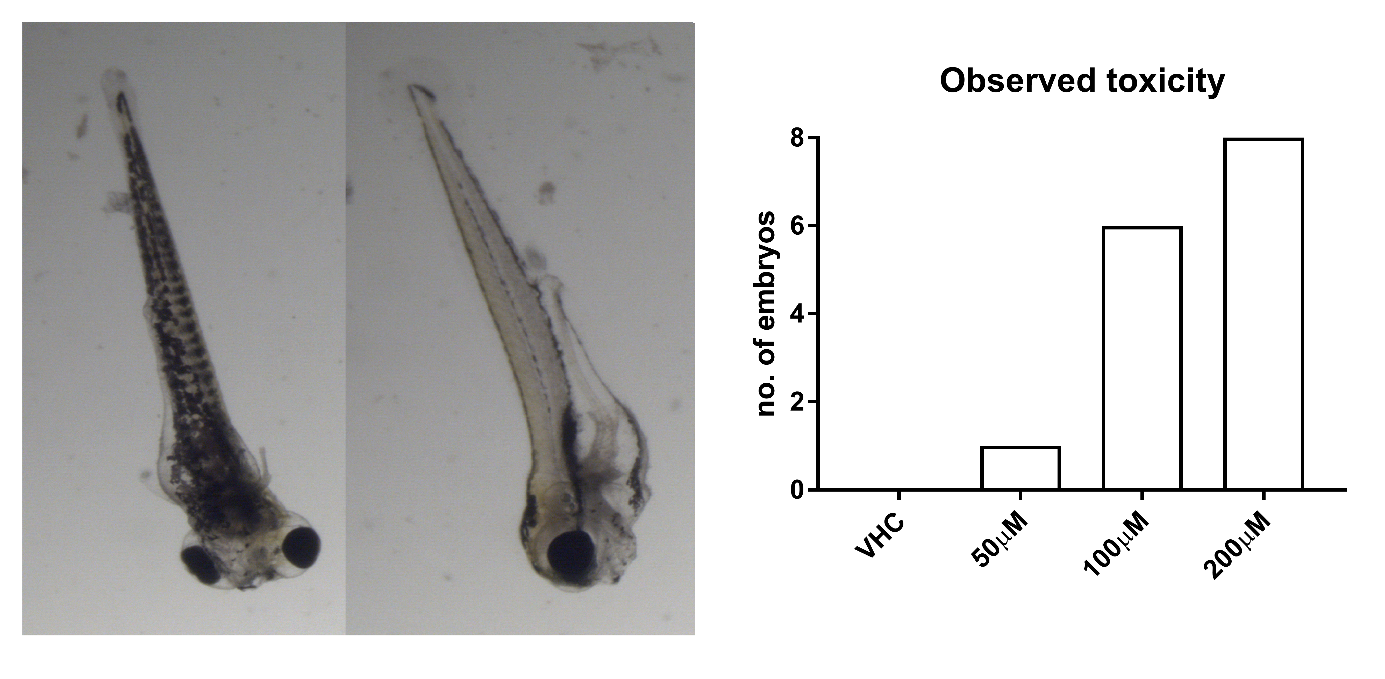


**S6 Clemizole toxicity after long-term exposure** *Scn1Lab* embryos exposed to 100µM or 200µM clemizole showed toxicity after 24h incubation including malformations and death.50µM Clemizole was used instead of 100µM for AED exposure experiments.
