# Supplementary material for: NaV1.1 and NaV1.6 selective compounds reduce the behavior phenotype and epileptiform activity in a novel zebrafish model for Dravet Syndrome
Source: PLoS One. 2020 Mar 5;15(3):e0219106. doi: 10.1371/journal.pone.0219106 (PMC7058281; doi:10.1371/journal.pone.0219106)
Supplement: S1 Table — (DOCX) [file pone.0219106.s009.docx]

**Table S1** Oligos

| Name | Seqeunce | Info |
| --- | --- | --- |
| sgRNA | ATTTAGGTGACACTATAGATTGGTTGGCAGGTTGCCGGTTTTAGAGCTAGAAATAGCAAG | SP6 promoter, gene specific oligo and overlap oligo |
| Constant oligo | AAAAGCACCGACTCGGTGCCACTTTTTCAAGTTGATAACGGACTAGCCTTATTTTAACTTGCTATTTCTAGCTCTAAAAC | Cas9 recognition site |
| Primer1 | CTTCGGCGCTCACTGTTT | Amplify KO region |
| Primer2 | CGGCTTTCTCCTCTTCCTCT |  |
| Primer3 | GCCACCACAATCCAAAAGGT | chr2:21162581-21162603 |
| Primer4 | GGCACTTTTGAAGACCCACT |  |
| Primer5 | AGAAGGGACATGAACAGGCA | chr3:38537484-38537506 |
| Primer6 | CCAACCATCCGATCAATGCC |  |
| Primer7 | CAGGAATGGAGTGTGAGCGT | chr7:4810353-4810375 |
| Primer8 | TCCGTCATTCAGCACATCACA |  |
| Primer9 | ATAGAGGTGGGTGTGGAGCT | chr7:4815293-4815315 |
| Primer10 | GCTCTCATCCGTCATTCAGC |  |
| Primer11 | AAGTTCGCCTTTGAGCCTCA | chr17:20571818-20571840 |
| Primer12 | TCACCCTGCTTTGACTGAACT |  |
